# A Pilot Study of CPR Quality Comparing an Augmented Reality Application vs. a Standard Audio-Visual Feedback Manikin

**DOI:** 10.3389/fdgth.2020.00001

**Published:** 2020-02-28

**Authors:** Marion Leary, Shaun K. McGovern, Steve Balian, Benjamin S. Abella, Audrey L. Blewer

**Affiliations:** ^1^Center for Resuscitation Science and Department of Emergency Medicine, University of Pennsylvania, Philadelphia, PA, United States; ^2^School of Nursing, University of Pennsylvania, Philadelphia, PA, United States; ^3^Department of Family Medicine and Community Health, Duke University, Durham, NC, United States

**Keywords:** cardiopulmonary resuscitation, augmented reality, gaming, simulation, cardiac arrest

## Abstract

**Background:** Guidelines-based cardiopulmonary resuscitation (CPR) during in-hospital cardiac arrest is a significant predictor of survival, yet the quality of healthcare provider (HCP) CPR (e.g., nurses, physicians etc.) has been shown to be poor. Studies have found that providing HCPs with simulated CPR refresher trainings can improve their CPR quality, however, no studies have compared the use of an augmented reality (AR) CPR refresher training with a standard audio-visual (AV) feedback manikin to improve HCP training.

**Objectives:** In our pilot study, HCPs were randomized to a refresher CPR simulation training with either our AR CPR training application (CPReality) or a standard AV feedback manikin. All subjects completed 2 min of CPR on their respective CPR training modalities, followed by an additional 2 min post-simulation CPR evaluation with no feedback. We hypothesized that the AR CPR training application would confer improved CPR quality defined as chest compression rate and depth compared with the standard AV feedback training.

**Results:** Between January 2019 and May 2019, 100 HCPs were enrolled (50 in the CPReality cohort and 50 in the standard AV manikin cohort). The mean chest compression (CC) rate for all subjects during the intervention was 118 ± 15 cpm, and CC depth was 50 ± 8; post-intervention the CC rate was 120 ± 13 and CC depth was 51 ± 8. The mean CC rate for those trained with CPReality was 121 ± 3 compared with the standard CPR manikin training which was 114 ± 1 cpm (*p* < 0.006); CC depth was 48 ± 1 mm vs. 52 ± 1 (*p* = 0.007), respectively. Post-simulation CPR quality with no feedback showed a mean CC rate for the CPReality application at 122 ± 15 cpm compared with the standard CPR manikin at 117 ± 11 cpm (*p* = 0.09); depth was 49 ± 8 mm vs. 52 ± 8 (*p* = 0.095), respectively. In the post-survey, 79% of CPReality subjects agreed that the AR application provided a realistic patient presence compared with 59% (*p* = 0.07) of subjects in the standard CPR manikin cohort.

**Conclusions:** In a randomized trial of an AR CPR training application compared with a standard CPR manikin training, the AR CPR application did not improve the quality of CPR performed during a CPR refresher training compared with the standard training in HCPs. Future studies should investigate the use of this and other digital technologies for CPR training and education.

## Introduction

Cardiac arrest occurs when the heart suddenly ceases its normal activity of circulating blood throughout the body. In-hospital cardiac arrest (IHCA) affects over 290,000 adults annually in the United States, with <25% surviving to hospital discharge (1, 2). Survival from IHCA has been shown to be associated with resuscitation processes such as guidelines-based cardiopulmonary resuscitation (CPR) (3–6). While guideline-based CPR has been shown to be a significant contributor to survival, numerous studies have found that CPR quality performed by healthcare providers (HCPs), for example, nurses and physicians, is often outside of guideline recommendations, with shallow chest compression (CC) depths and variable CC rates (3–6).

The American Heart Association (AHA) released a scientific statement on educational strategies to improve outcomes from cardiac arrest (7); highlighted within that statement was the need to consider more innovative solutions for CPR training and education, including the use of digital strategies such as Augmented Reality (AR). AR is a computer-generated holographic image that is overlaid into the real environment, allowing the user to interact with both the hologram and real objects in an integrated fashion. AR is a relatively new technology that is in its infancy for training and education in healthcare. A recent scoping review of the use of AR for medial application found that it has the potential to be a feasible application for training and education of providers (8). In resuscitation, few studies have examined its efficiency and efficacy in improving CPR quality. One study which examined the use of AR in a simulated pediatric resuscitation scenario found that it did not confer improved pediatric advanced life support (PALS) skill performance compared with a standard PALS pocket card (9).

AR immersion has the potential to improve CPR training in HCPs, allowing for the visual emphasis of the high-quality circulation of blood flow to the brain and other vital organs during resuscitation, positively disrupting the current CPR training paradigm. Whether using AR to increase visual learning during CPR training for HCPs would improve guidelines-quality CPR compared with a standard CPR training is unknown. Therefore, as a pilot study, we sought to examine the use of an AR CPR training application compared with a standard CPR training manikin to determine if the AR technology could improve HCP CPR quality defined as chest compression rate and depth.

## Materials and Methods

### Study Design

Using established AR technology, we created a hands-only (no mouth-to-mouth ventilation) CPR training application, CPReality, which integrated a CPR feedback manikin (Laerdal ResusciAnne; Laerdal Medical, Wappinger Falls, NY) with the Microsoft HoloLens (Microsoft, Redmond WA). The current pilot study was designed to establish the protocol, assess feasibility, test the interventions, and establish performance measures. In this partially-blinded randomized controlled trial (subjects did not know what intervention they would be randomized to but the person performing the data analysis was not blinded against the intervention), we sought to compare the AR CPR system with a standard CPR feedback manikin in HCPs during a simulated CPR refresher training. We hypothesized that the AR application would improve CPR skills compared with a standard CPR feedback manikin.

### Participant Selection

In this pilot study, we performed a randomized controlled trial among HCPs from our health care system. The randomization module in REDCap v9.5.3, Vanderbilt University, Nashville, TN; (10) was utilized to create a randomization table that equally balanced between interventions. Subjects were randomized on the individual-level at time of enrollment, and survey packets were pre-labeled based on the randomization scheme to either the AR CPR arm or the CPR feedback manikin arm. HCPs were randomized to a refresher CPR training with either our novel AR CPR training application (CPReality) or a standard AV CPR feedback manikin training. A convenience sample of HCPs were approached on the clinical floors in the in-patient setting, at our simulation center, or school of nursing, by a trained research coordinator and asked to participate in the study. Our research coordinator set up the CPR training in available breakrooms in the locations where enrollment was occurring. HCPs were able to participate during their breaks in clinical care or prior to educational trainings. HCPs were randomized at the individual level using an online randomization scheme, which was assigned to the subject via the survey packets. The study was approved by the Institutional Review Board of the University of Pennsylvania and given exemption from written informed consent. Therefore, verbal consent was obtained from subjects prior to being randomized into the study. Inclusion criteria included any HCPs (nurse, physician, EMT, respiratory therapist etc.) who was physically capable of performing CPR. Exclusion criteria included anyone who did not identify as a HCPs, <18 years of age, and anyone unable to perform CPR.

### CPReality

**Figure 1:**
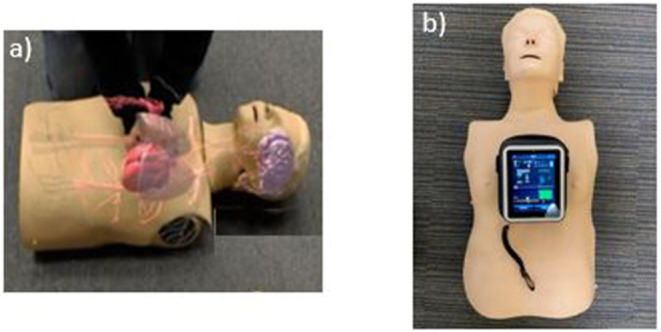
**(a)** AR CPR training application (CPReality); **(b)** standard CPR feedback training manikin.

The CPReality system was created in 2017 at the University of Pennsylvania for an internal technology application competition. CPReality is a novel AR system that integrates the Microsoft Hololens, with a CPR feedback manikin to create a holographic image of the human circulatory system (Figure 1a). As the trainee performs CPR on the feedback manikin, data are rendered into the Microsoft HoloLens and a holographic image of the circulatory system is overlaid next to the feedback manikin. CPReality was responsive to the performance of the subjects, as the blood flow to the brain increased or decreased based on the quality of the CPR they performed. Subjects could visualize the blood flow, as well as hear an audio heartbeat metronome, which increased or decreased depending on the actual quality of their CPR performed on the feedback manikin (Link to video: https://www.youtube.com/watch?v=QfiP62A-2qk). The CPR reality application was described in a previous publication and CPR quality metrics were reported (11).

### Standard CPR

The standard CPR training application included a CPR manikin with a validated AV feedback system (Laerdal Medical, Wappinger Falls, NY). The feedback device incorporated visual and audio cues on an accompanying computer tablet that trainees can use to improve the quality of their CC rate and CC depth in real time. The visual cues showed an increase or decrease in CC rate with a speedometer icon and CC depth with a bar chart that highlighted the optimal depth with a horizontal line (Figure 1b). The rate and depth icons increased or decreased based on the quality of CPR being performed by the subject on the feedback manikin.

### Study Protocol

Both cohorts (CPReality and standard) completed 2 min of CPR with their respective forms of AV feedback, along with a pre-and post-survey. No additional CPR feedback was provided to the subjects by our study personnel between the simulation and testing sessions. Immediately following the completion of the post-survey (8 ± 7 min), all subjects performed an additional 2 min of CPR on a CPR recording manikin with no feedback.

### CPR Skills Data

Similar to metrics set by the AHA Guidelines and Consensus Statements, the CPR skills data captured hands-only CPR metrics including mean CC rate, CC depth and CC fraction (12, 13). Guideline quality CPR for both cohorts was set at the AHA recommended guidelines of: CC rate of 100–120 compressions per min (cpm) and CC depth of 50 and 60 millimeters (mm). Compressions performed to proper depth and compressions performed at proper rate were also captured. Average compression rate over 2-min was reported. CC fraction was the percentage of CC performed over the 2- min compression period. CPR quality data were captured from the CPR feedback manikin and downloaded as a.csv file, which was imported into the statistical software package for analysis.

### Pre-and Post-survey

Pre-and post-survey questions were completed by both cohorts of subjects (Data Sheet 1–3) using paper data collection forms. These survey data were then input into our RedCAP database. The pre-survey collected information including: demographic data (age, gender, race) as well as healthcare position (Nurse, Physician, Respiratory Therapist etc.), years in practice, and time since last re/certification in basic life support. The post-survey collected quantitative, Likert-scale responses as well as free-text qualitative responses related to the use of AR in general, as well as specific questions about its use during the scenario.

### Statistical Analysis

A standard statistical software package was used to analyze the data (STATA 12, Statacorp, College Station, TX). As our study was designed to show a difference between the two cohorts the significance level was set at the standard threshold of 0.05, with a difference declared at a *p* < 0.05. Descriptive statistics were used to report subject demographic and characteristic data. Student's *t*-test was used to compare the CPR quality of the two cohorts, as well as pre-and post-survey data. Chi-squared test was used for proportion data, and Wilcoxon rank-sum test was used for non-parametric equality testing of medians. The Shapiro-Wilks test for normality was performed. As this was a pilot study, we did not have adequate power to detect a difference between cohorts. The results of this study will be used to perform a sample size calculation for a future larger randomized trial.

## Results

Between January 2019 and May 2019 100 HCPs (e.g., nurses, doctors, Advanced Practice Nurses) were enrolled. Fifty subjects were randomized to the CPReality cohort and 50 subjects were randomized to the standard AR feedback manikin cohort. Mean age was 37 ± 12 years, 77 (77%) were female, and 81 (81%) were Nurses or Advanced Practice Nurses. The mean years of healthcare experience were 12 ± 11 years (Table 1). In the AR cohort, 38 (76%) were female and 39 (88%) were female in the standard cohort (*p* = 0.8). Of the subjects in the AR cohort, 40 (80%) were Nurses compared with 41 (82%) in the standard cohort (*p* = 0.3). Mean years of experience per cohort was 11 ± 10 years for the AR group and 13 ± 11 years for the standard AV feedback group (Table 1).

**Table 1:** Subject demographics.

***n* = 100**	**Total**	**CPReality**	**Standard**
		***n* = 50**	***n* = 50**
Age, yrs (m ± sd)	37 ± 12	35 ± 11	39 ± 13
Gender, *n* (%)			
Female	77 (77)	36 (76)	39 (78)
Race/Ethnicity, *n* (%)			
Asian	7 (7)	6 (12)	1 (2)
Black	8 (8)	3 (6)	5 (10)
Hispanic/latino	1 (1)	0 (0)	1 (2)
Other	1 (1)	0 (0)	1 (2)
White	83 (83)	41 (82)	42 (84)
Healthcare Provider, *n* (%)			
Advanced practice nurse	9 (9)	5 (10)	4 (8)
Nurse	72 (72)	35 (70)	37 (74)
Other	9 (9)	6 (12)	5 (6)
Physician	3 (3)	1 (2)	2 (4)
Physician assistant	2 (2)	0 (0)	2 (4)
Respiratory therapist	5 (5)	3 (6)	2 (4)
Time since last BLS recertification, *n* (%)			
<1 month	10 (10)	5 (10)	5 (10)
2–6 months	21 (21)	9 (18)	12 (25)
7–12 months	32 (32)	17 (36)	14 (28)
13–24 months	24 (24)	12 (24)	12 (25)
>24 months	7 (7)	(8)	3 (6)
Not certified	4 (4)	2 (4)	2 (4)
No response	2 (2)	0 (0)	1 (2)

*N, number; yrs, years; m, mean; sd, standard deviation; BLS, basic life support*.

Data for CPR quality (CC rate and CC depth) were normally distributed in both testing phases with the exception CC rate in the *post*-testing phase (*p* = 0.03), therefore, supplemental data showing median and interquartile ranges have been included (Table S1). The mean CC rate for all subjects enrolled during the initial simulation testing was 120 ± 13 cpm, CC depth was 50 ± 8 mm and CC fraction was 98 ± 5 percent. Post-simulation the overall mean CC rate was 120 ± 13 cpm, CC depth was 51 ± 8 mm, and CC fraction was 99 ± 4%.

**Figure 2:**
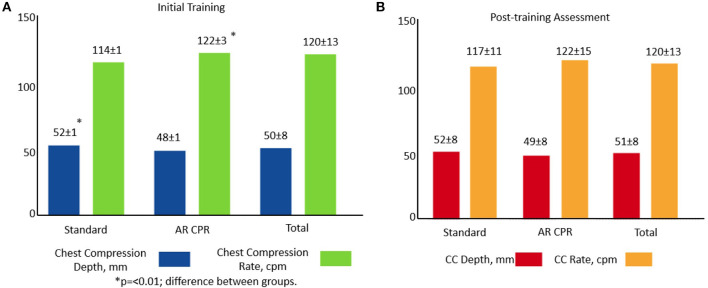
CC rate and depth results by training modality: **(A)** initial and **(B)** post-simulation.

When comparing the standard feedback manikin with the AR application during the initial training session, the mean CC rate was 114 ± 1 cpm vs. 122 ± 3 cpm, (*p* = 0.007) and CC depth was 52 ± 1 mm vs. 48 ± 1 mm (*p* = 0.006), respectively. Post-simulation, the mean CC rate for the standard feedback manikin compared with the AR application was 117 ± 11 cpm vs. 122 ± 15 cpm vs. (*p* = 0.09) and depth was 52 ± 8 mm vs. 49 ± 8 mm (*p* = 0.095), respectively (Figure 2). There was no difference in CC fraction during either testing session (data not shown).

Of all subjects, just 28/98 (29%) had both CC rate and depth within AHA guideline recommendations during the initial simulation and 25/96 (26%) in the post-simulation testing session. When comparing between groups, in the initial simulation session, 23/49 (47%) of subjects in the standard manikin compared with 5/49 (10%) of subjects in the AR cohort, had CC within both guideline rate and depth (*p* < 0.001). In the post-simulation testing session, 17/47 (36%) of subjects in the standard manikin cohort compared with 8/49 (16%) of AR subjects had both guideline rate and depth (*p* = 0.03).

**Figure 3:**
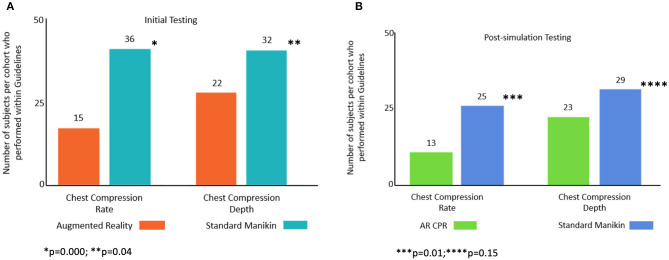
Number of subjects per cohort that performed CC rate and CC depth within guideline ranges: **(A)** initial and **(B)** post-simulation.

When comparing the proportion of subjects who met AHA Guidelines for CC rate during the initial simulation testing phase, in the standard manikin cohort 36/49 (74%) of subjects were within guidelines compared with 15/49 (31%) of the AR subjects (*p* = 0.000); for CC depth 32/49 (65%) of the standard manikin cohort were within guidelines compared with 22/49 (45%) of AR subjects (*p* = 0.04). During the post-simulation testing phase for CC rate, 25/47 (53%) of standard manikin subjects were within guidelines compared with 13/49 (27%) of AR subjects (0.01); for CC depth, 29/47 (62%) of the standard manikin cohort were within guidelines compared with 23/49 (47%) in the AR cohort (*p* = 0.15, Figure 3).

In the post-survey, of those who completed it, 34/43 (79%) of AR subjects agreed that the AR simulation provided a realistic patient presence compared to 16/27 (59%; *p* = 0.07) of subjects in the standard feedback manikin cohort. The majority of AR simulation subjects, 40/42 (95%), agreed or strongly agreed that they would want to use the AR modality for future CPR trainings, while 43/43 (100%) of subjects who completed the survey agreed or strongly agreed that the visualization of blood flow was useful.

## Discussion

In our pilot study comparing our novel AR CPR training system with a standard AV feedback manikin during a CPR refresher training, the AR system did not confer better CPR skills overall, as measured by average CC rate and depth during post-simulation testing. When examining the proportion of subjects who performed CC rate and CC depth within guidelines, the subjects in the AR CPR training system had poorer CPR metrics compared to the standard feedback manikin, however, both cohorts had extremely low percentages of CC rate and depth within recommended guidelines. When examining the mean CPR quality data the AR system was just slightly outside of guidelines with the CC rate of 1 to 2 cpm over guidelines recommendation and a depth of 1 to 2 mm below the guidelines recommendations, which contributed to the significant difference in the proportion of subjects in each cohort who were outside of AHA guideline recommendations. This could be due to a design flaw with the AR system; as this was a pilot study to test feasibility, these results will be taken into consideration when considering how to upgrade the CPReality application for better CPR feedback.

Ultimately just a small percentage of either HCP group were within guidelines for CC rate and depth, which shows that as a resuscitation education community we need to consider different ways to keep HCP CPR skills up-to-date. This is an issue that has been reported on for over a decade, as was reported in the Journal of American Medical Association in 2005 (14, 15). In the in-hospital setting, HCP CPR is vital to patient survival from cardiac arrest, but often the quality of CPR provided falls below recommended guidelines in both rate and depth (14, 15). While HCPs are required to recertify in basic life support (BLS) regularly (i.e., every 2 years), studies have found skill decay occurs after just a few months (16). Though CPR was established as the method of cardiac and respiratory resuscitation over fifty years ago, standard CPR training courses have not readily adapted current digital technologies that could enhance these skills. In general HCPs who are trained in CPR attend an in-person course where they watch CPR training videos and are taught CPR skills by instructors on a CPR training manikin; the trainees then perform a skills test on the manikin and complete a written knowledge test. While groups have worked to innovate this standard CPR training with online courses, and virtual video trainings, these modalities do not visually emphasize the importance of high-quality CPR for the circulation of blood flow to the brain and other vital organs during resuscitation.

Studies have found that the quality of CPR and survival are significantly improved when providers guide their CPR in conjunction with physiology (17). Whether using an AR CPR training application that allows for the visualization of human physiology with the adaption of psychomotor skills to improve the quality of CPR being performed by HCPs is unknown. Our findings suggest that subjects were able to adapt to the AR CPR training environment and improve their cohort CPR quality overall, though it was not better than the standard manikin training, and subjects performed within guidelines range significantly less. Larger studies should be performed to determine if AR CPR training could improve overall CPR quality for new and re-certifying HCPs.

The use of AR during cardiac arrest and for CPR training is nascent, with very few studies applying these technologies to the resuscitation field. One study that has leveraged the use of AR technology to examine adherence to the pediatric advanced life support (PALS) guidelines, as established by the AHA, compared the use of AR glasses with a standard PALS pocket card during simulated resuscitation. They found that the application of AR glasses did not improve resuscitation metrics such as time to first defibrillation attempt compared with PALS pocket cards, but adherence to dosing of the defibrillator was improved (9). Our team examined the feasibility of using the AR system to capture CPR metrics in HCPs and found that HCPs were able to provide high quality CCs using the AR CPR system, and that most subjects would allow for consideration of the use of this type of technology for CPR training (11).

The majority of subjects in the AR CPR cohort stated that the patient visualization was more realistic and stated that the physiologic holographic images were beneficial to their training. There are limited studies that examine how increasing realism during resuscitation impacts HCP CPR quality, however if HCPs are able to visualize the real-time physiological response to the quality of their CPR, such as blood flow to the brain and other vital organs, it could translate into better quality CPR in real life. Although past studies have found that AV feedback can improve CPR quality (18), the additional application of the AR technology to overlay a holographic image of the circulatory system, which is responsive to the quality of CPR being performed, is novel. While studies have found that using immersive technologies similar to AR, such as virtual reality, can improve trainee experience and confidence, to our knowledge, no studies have linked these technologies with outcomes (19–22).

In addition to the holographic component of CPReality, the AR CPR system also featured a gamification approach, where subjects controlled the blood flow to the brain by increasing or decreasing the quality of the CPR being performed. Serious games, which incorporate the application of gaming relating to an educational topic, such as CPR, have been used in resuscitation in recent years. One study of the use of gaming for pediatric education of medical providers found that it allowed HCPs to train without risk (23). Another study examined the use of gamification compared with a standard online course for medical students and found that the serious game was not superior for cardiac arrest management (24). In addition, that examined the gamification literature found that focused use of gamification has the ability to increase learner engagement and enthusiasm thus aligning with learning goals (25). Finally, one study which examined the use of gamification for critical pediatric conditions including respiratory failure and supraventricular tachycardia found that gamification can improve written test scores (26).

Though few studies examine the use of AR for resuscitation simulation, a larger body of evidence exists using virtual reality (VR) for CPR training, as well as for observation of bystander response metrics, and other resuscitation skills (20, 22, 27–32). Due to the considerable difference in VR from AR, whereas in the VR environment a user cannot see or interact with the real environment, comparing studies using VR may not be relevant. Nevertheless, these studies show that there is a growing interest in integrating technology to improve training and education for CPR and resuscitation training.

## Limitations

There are a number of limitations inherent in our study. The study recruited a convenience sample of HCPs from two hospitals and two training centers, within our university health system and in close proximity, therefore selection bias of HCPs may have occurred. Additionally, HCPs who participated in the study may have had higher CPR self-efficacy. We were not able to capture CPR skill data outside of the immediate training day, and therefore were not able to assess skill decay; whether the CPReality system would have improved CPR skill retention is therefore unknown. As the use of AR technology is relatively new in the healthcare setting, there may have been a learning bias in the cohort that was randomized to CPReality, affecting the subjects' CPR quality. As our study was only examining CPR quality, we do not know if knowledge retention would be increased due to the use of the CPReality system. Finally, as this was a pilot study, our sample size was small and may not be large enough to detect difference in CPR quality between cohorts.

## Conclusions

In the randomized trial of an AR CPR training system (CPReality) compared with a standard AV feedback CPR training, the AR CPR simulation training produced similar CPR quality overall post-simulation in HCP, but significantly more subjects performed outside of both guideline ranges for CC rate and depth. Determining if technology-enhanced CPR training modalities can improve CPR quality is an important step to improving patient outcomes and one that requires further study.

## Data Availability Statement

The datasets generated for this study are available on request to the corresponding author.

## Ethics Statement

The studies involving human participants were reviewed and approved by University of Pennsylvania Institutional Review Board. Written informed consent for participation was not required for this study in accordance with the national legislation and the institutional requirements.

## Author Contributions

ML, BA, and AB conceived and designed the study. ML, SM, SB, and AB contributed to data collection and/or data analyses. ML, SM, SB, BA, and AB assisted with the writing of the manuscript.

### Conflict of Interest

ML has received research support from the Zoll Foundation for this work. ML has received research support from the American Heart Association. ML has received in-kind support from Laerdal Medical. ML has licensed intellectual property for virtual reality CPR applications. BA has received research support from the NIH NHLBI, PCORI, the Medtronic Foundation, Physiocontrol and the American Heart Association. BA has received honoraria from CR Bard and Physio-Control. BA is an advisor for MDAlly. The remaining authors declare that the research was conducted in the absence of any commercial or financial relationships that could be construed as a potential conflict of interest.
