# Supplementary material for: A Pilot Study of CPR Quality Comparing an Augmented Reality Application vs. a Standard Audio-Visual Feedback Manikin
Source: Front Digit Health. 2020 Feb 28;2:1. doi: 10.3389/fdgth.2020.00001 (PMC8521903; doi:10.3389/fdgth.2020.00001)
Supplement: Supplementary file 1 [file Data_Sheet_1.pdf]

Instructions: *This survey is designed to gather data for research purposes only. All data is confidential and de-identifiable.*

Date: m: / d: / y:
Subject Number:
RA Initials: Site:

Pre-Training Survey

Age: ________

Gender:

☐ Male ☑ Female ☐ Other ____________

Race:

☐ White ☐ Black ☐ Hispanic/Latino ☐ Asian/Pacific Islander ☐ Other____________

Healthcare Position:

☐ Nurse ☐ Tech ☑ Respiratory Therapist ☐ PT/OT ☐ Physician Assistant

☐ APRN (MSN, DNP, etc.) ☐ Physician ☐ Pharmacy ☐ Other ____________

Hospital:

☐ HUP ☑ Presby ☐ Pennsy ☐ Chester County ☐ Other ____________

Hospital Floor/Unit: ____________________________

How many years of healthcare experience do you have? ________ ________

How long ago did you last re/certify in BLS?

☐ <1 month ☑ 2-6 months ☐ 7-12 months ☐ 13-24 months ☐ >24 months ☐ Not Certified

How long ago did you last re/certify in ACLS?

☐ <1 month ☐ 2-6 months ☐ 7-12 months ☐ 13-24 months ☐ >24 months ☑ Not Certified

On average, how many cardiac resuscitations are you involved in per month?

☐ 0 ☐ 1 ☐ 2-3 ☐ 4-5 ☑ >5
