# Supplementary material for: A Pilot Study of CPR Quality Comparing an Augmented Reality Application vs. a Standard Audio-Visual Feedback Manikin
Source: Front Digit Health. 2020 Feb 28;2:1. doi: 10.3389/fdgth.2020.00001 (PMC8521903; doi:10.3389/fdgth.2020.00001)
Supplement: Supplementary file 2 [file Data_Sheet_2.pdf]

Instructions: *This survey is designed to gather data for research purposes only. All data is confidential and de-identifiable.*

Date: m: / d: /y:
Subject Number:
RA Initials: Site:

Post-Training Survey

Have you used a wearable augmented reality device in the past?:

☐ Yes ☐ No

I was comfortable using the augmented reality device.:

☐ Strongly Agree ☐ Agree ☐ Disagree ☐ Strongly Disagree

I had the feeling that the patient was really present in front of me.

☐ Strongly Agree ☐ Agree ☐ Disagree ☐ Strongly Disagree

The experience of visualizing the blood flow of the augmented reality victim while performing CPR was useful.

☐ Strongly Agree ☐ Agree ☐ Disagree ☐ Strongly Disagree

I want to use this augmented reality CPR application for training in the future.

☐ Strongly Agree ☐ Agree ☐ Disagree ☐ Strongly Disagree

Please tell us what you liked about the augmented reality CPR training application? (answer in the space below)

What would you change about the augmented reality CPR training application? (answer in the space below)
