# Supplementary material for: A Pilot Study of CPR Quality Comparing an Augmented Reality Application vs. a Standard Audio-Visual Feedback Manikin
Source: Front Digit Health. 2020 Feb 28;2:1. doi: 10.3389/fdgth.2020.00001 (PMC8521903; doi:10.3389/fdgth.2020.00001)
Supplement: Supplementary file 3 [file Data_Sheet_3.pdf]

Instructions: *This survey is designed to gather data for research purposes only. All data is confidential and de-identifiable.*

Date: m: / d: /y:
Subject Number:
RA Initials: Site:

Post-Training Survey

I had the feeling that the patient was really present in front of me.

☐ Strongly Agree ☐ Agree ☐ Disagree ☐ Strongly Disagree

Please tell us what you liked about the CPR manikin training? (answer in the space below)

What would you change about the CPR manikin training? (answer in the space below)
