# Supplementary material for: A Pilot Study of CPR Quality Comparing an Augmented Reality Application vs. a Standard Audio-Visual Feedback Manikin
Source: Front Digit Health. 2020 Feb 28;2:1. doi: 10.3389/fdgth.2020.00001 (PMC8521903; doi:10.3389/fdgth.2020.00001)
Supplement: Supplementary file 4 [file Table_1.docx]

**Supplementary Table 1**. Chest compression (CC) rate and depth between cohorts.

| n=100 | Total | CPReality | Standard |
| --- | --- | --- | --- |
|  |  | n=50 | n=50 |
| Simulation testing, m(IQR) |  |  |  |
| CC rate, cpm | 119 (68,157) | 124 (112, 135) | 114 (107, 120)* |
| CC depth, mm | 51 (30, 64) | 47 (42, 54) | 52 (47, 57) |
| Post-simulation testing |  |  |  |
| CC rate, cpm | 121 (73, 157) | 124 (117, 130) | 119 (109, 125)** |
| CC depth, mm | 51 (29, 63) | 49 (45, 56) | 53 (48, 59) |

CC, chest compression; m, median; IQR, interquartile range; cpm, chest compressions per minute; mm, millimetre; %, percent; *p=0.001;**p=0.014
